# Scale-dependent effects of habitat fragmentation on reproduction in the annual *Circaeaster agristis*, a narrow endemic and threatened species

**DOI:** 10.1186/s40529-015-0095-5

**Published:** 2015-06-02

**Authors:** Jie-Cai Zhao, Jun Luo, Chun-Ping Yang, Guo-Xing Cao

**Affiliations:** 1 Department of Forestry, Sichuan Agricultural University, Yaan, 625014 China; 2 Xishuangbanna Tropical Botanical Garden, Chinese Academy of Sciences, Mengla, 666303 China

**Keywords:** Circaeaster agristis, Habitat fragmentation, Population size, Population density, Reproduction, Scale-dependency

## Abstract

**Background:**

Habitat fragmentation and the resulting decline in population size and density commonly reduce the reproduction of rare and threatened species. We investigated the impacts of population size and density on reproduction in more than 30 populations of *Circaeaster agristis*, a narrow endemic and threatened species, in 2010 and 2011. We also examined the effects of NND (nearest neighbor distance) and LNS (local neighbor size), within radii of 0.1 m, 0.2 m and 0.3 m, on reproduction in two of the populations in 2011.

**Results:**

Population size did not affect fruit (seed) number and fruit set in either year studied. Population density had an indirect negative effect on fruit number and fruit set as a consequence of a negative effect on plant size in 2010, but had no effect on fruit number and fruit set in 2011. Within populations, individual fruit number did not change, and individual fruit set increased independent of plant size, in response to increasing NND. Both individual fruit number and individual fruit set increased, independent of plant size, with increases in LNS within a 0.1 m radius, but did not change with increases in LNS within radii of between 0.1 m and 0.2 m radii or between 0.2 m and 0.3 m.

**Conclusions:**

The effect of habitat fragmentation on reproduction of *C. agristis* is scale-dependent. In contrast to the generally accepted idea that fragmentation reduces plant reproduction, reproductive success may increase in sparse populations or increase in response to decreases in LNS in *C. agristis*.

**Electronic supplementary material:**

The online version of this article (doi:10.1186/s40529-015-0095-5) contains supplementary material, which is available to authorized users.

## Background

Habitat fragmentation is a worldwide phenomenon, and is considered to be one of the major threats to the persistence and viability of plant populations (Eriksson and Ehrlén [2001]; Oostermeijer, [2003]). Habitat fragmentation often causes a decline in the size and density of populations, thereby altering abiotic and biotic environmental conditions (Wilcove et al., [1986]; Saunders et al., [1991]), all of which can affect the reproductive success of plants.

Experimental studies and field observations have shown that plants in small and sparse populations, because of their smaller display size and lower supply of rewards, often receive lower visitation rates (Ågren, [1996]; Kunin, [1997]; Weber and Kolb [2013]), and experience smaller pollen loads (Bosch and Waser, [1999]; Waites and Ågren, [2004]; Jakobsson et al., [2009]), resulting in a limitation of pollen quantity and reduced production of fruits and seeds. Furthermore, plants in small and sparse populations may witness higher within-plant movement of pollinators (Antonovics and Levin, [1980]; Klinkhamer and de Jong, [1990]; Hermansen et al., [2014]), or may be more likely to cross with related individuals (Barrett and Kohn, [1991]; Glémin et al. [2008]; Suarez-Gonzalez and Good, [2014]). In self-incompatible plants, within-plant pollinator movements or crossing with related individuals decrease compatible pollen receipt, which can reduce reproductive success (Waites and Ågren, [2004]). In self-compatible plants, within-plant pollinator movements or crossing with related individuals lead to greater self-fertilization rates (van Treuren et al., [1993]; Karron, [1995]) or biparental inbreeding (Jones and Comita, [2008]), potentially decreasing offspring quantity or quality. Finally, if the population size and density are positively correlated with habitat quality, plant size and reproductive success may increase in response to increasing population size and density (Leimu et al., [2006]). Literature reviews on this topic have indicated that a positive correlation between reproductive success and population size or density is a common phenomenon in rare and endangered species (Ghazoul, [2005]; Leimu et al., [2006]).

Notwithstanding the above points, pollination and reproductive success may not always be curtailed in small and sparse populations. For example, plants in such populations may experience lower competition for pollinator visits than those in large and dense populations (Steven et al., [2003]). If fruit or seed set is pollinator-limited and pollinators visit a smaller proportion of flowers per plant in large and dense rather than small and sparse populations, then reproductive success may decline in large and dense populations, particularly in self-incompatible species (Johnson et al., [2012]; Stein et al., [2013]; Ward et al., [2013]). Plants facing chronic pollinator scarcity and pollen limitation may evolve self-compatibility for reproductive assurance (Baker, [1967]), and are expected to be less subject to reduced pollinator visitation and the effects of fragmentation (Aguilar et al., [2006]). Furthermore, reproductive success in plants in sparse populations may benefit from lower competition for resources, such as light, soil nutrients and water. As a result, plant size and reproductive success may decrease with plant density (Harper, [1977]; Antonovics and Levin, [1980]; Weiner, [1982]; Mustajärvi et al., [2001]).

When assessing the effects of habitat fragmentation on reproductive success, some researchers have focused on the population level: population size (number of individuals) and/or mean population density (Ågren, [1996]; Morgan, [1999]; Hermansen et al., [2014]). On the other hand, others have focused on the individual level: nearest neighbor distance (NND) (Allison, [1990]; Metcalfe and Kunin, [2006]; Caraballo-Ortiz et al., [2011]; Lawes et al., [2013]) or local neighbor size (LNS) (Roll et al., [1997]; Mitchell and Ankeny, [2001]; Jakobsson et al., [2009]; Weber and Kolb [2013]). Although the perspective from either level can provide valuable insight into the effects of habitat fragmentation, few studies have investigated the effects of habitat fragmentation on reproductive success at both levels within a single species (Wagenius, [2006]; Gunton and Kunin, [2007]; Spigler and Chang, [2008]).

In this study, we explored the effects of habitat fragmentation on reproductive success in the endangered annual *Circaeaster agristis*, at both population (population size and density) and individual (NND and LNS) levels, in 2010 and 2011. The data obtained here may assist in understanding the effects of fragmentation on plant reproductive success, and provide useful guidelines for the management and conservation of *C. agristis*.

## Methods

### Study species

*Circaeaster agristis* Maxim.is the only species of the genus *Circaeaster*, which is a member of Circaeasteraceae together with another monotypic genus, *Kingdonia* (Tian et al., [2007]). *Circaeaster agristis* is an annual alpine herb, narrowly distributed in southwestern and northwestern China. This species primarily grows in humus-rich forest soils. Its population is declining and its distribution range is shrinking because of deforestation and habitat fragmentation, and has consequently been listed as a rare and endangered species in the Chinese red list (Fu and Jin, [1992]). It reproduces exclusively by seeds. Individual plants of *C. agristis* produce a single stem which is, on average, 7.2 ± 2.2 cm (mean ± SD, range = 4.3-12.9 cm, *n* = 60, unpublished data) tall. The flowers are green, hermaphroditic and about 1 mm long, have two or three tepals, one or two stamens, and one carpel (Hu et al., [1990]). Flowering occurs from late May to early June, and fruits mature in August. The fruit is an elliptic achene, and is 6–12 mm long when mature. Each achene contains one seed. The upper surface of the fruit exhibits hooked trichomes.

### Study site

Our experiment was conducted in the Wanglang Nature Reserve (32°49′-33°02′ N, 103°55′-104°10′ E, 2300–4980 m above sea level), southwestern China. The vertical distribution of vegetation types in this region includes mixed forests of conifers and broadleaf trees, and broadleaf deciduous forest (2300–2600 m), fir forest (dominated by *Abies faxoniana*) and spruce-cypress forest (dominated by *Picea purpurea* and *Sabina saltuaria*, 2600–3500 m), subalpine shrubs and meadow (3500–4400 m), and sparse alpine vegetation (4400–4900 m).

### Population size and density

In 2010, we identified 34 *C. agristis* populations (Table 1). Three populations disappeared in 2011, but another four populations were identified in this year (Table 1). The populations selected for analysis were present at between 2503 and 2645 m altitude. All selected populations were present in a forest and were separated from one another by at least 30 m. Natural *C. agristis* populations are patchy and discrete, and are easy to delimit. In early to mid-August of each year, we measured the perimeter of each population, calculating its area, and then counted the number of individual plants (population size). We then estimated the mean density of each population by dividing its population size by its area. The log-transformed mean population density was significantly correlated to the log-transformed population size in 2010 (*r*_p_ = 0.371, *P* = 0.031, *n* = 34) and 2011 (*r*_p_ = 0.614, *P* < 0.001, *n* = 35).

**Table 1:** Summary data for study populations including location, population size and population density

Population	Location		Population size		Population density (number of plants/m^2^)	
	Latitude	longitude	2010	2011	2010	2011
1	32°58′32"	104°04′33"	312	93	118	38
2	32°58′35"	104°04′35"	15434	32647	230	211
3	32°58′05"	104°04′38"	25	7	71	28
4	32°58′44"	104°04′43"	18433	29277	146	122
5	32°58′42"	104°04′50"	8062	13961	129	122
6	32°58′42"	104°04′55"	715	253	63	21
7	32°58′42"	104°04′59"	138	—	139	—
8	32°58′39"	104°05′13"	1138	7761	27	117
9	32°58′14"	104°05′18"	257	31	612	20
10	32°58′34"	104°05′19"	5166	1905	118	43
11	32°58′28"	104°05′21"	2137	557	161	40
12	32°58′25"	104°05′24"	43495	22049	185	87
13	32°58′23"	104°05′26"	10224	7522	238	139
14	32°58′24"	104°05′28"	231	975	27	103
15	32°58′26"	104°05′30"	7231	1879	272	77
16	32°58′11"	104°05′37"	137	302	117	122
17	32°58′14"	104°05′43"	156	134	158	61
18	32°58′14"	104°05′48"	450	379	58	175
19	32°58′15"	104°05′54"	17	—	57	—
20	32°58′16"	104°05′59"	1315	7942	33	83
21	32°58′40"	104°05′08"	53	428	7	229
22	32°58′16"	104°06′09"	3860	247	283	11
23	32°58′16"	104°06′12"	37519	96071	309	469
24	32°58′09"	104°06′13"	3774	3340	165	178
25	32°58′12"	104°06′16"	8138	8961	134	36
26	32°58′07"	104°06′17"	301	—	1158	—
27	32°58′14"	104°06′17"	4912	4417	120	115
28	32°58′10"	104°06′20"	85897	91925	134	142
29	32°58′09"	104°06′24"	1537	147	422	21
30	32°58′09"	104°06′29"	13657	19962	184	89
31	32°58′02"	104°06′46"	84500	40357	285	133
32	32°57′59"	104°06′56"	18084	46203	239	275
33	32°58′00"	104°07′02"	247	554	30	71
34	32°57′58"	104°07′24"	426	1069	12	34
35	32°58′11"	104°03′18"	—	41389	—	88
36	32°58′46"	104°03′44"	—	91669	—	109
37	32°58′45"	104°03′57"	—	17157	—	70
38	32°58′29"	104°04′20"	—	70989	—	64

— the population disappeared in 2011 or not found in 2010

### Sampling on a population basis

From early to mid-August 2010 and 2011 in each population, 30 individuals were randomly marked, except in populations with less than 30 individuals, in which case all of them were marked. The number of leaves, flowers (flower scars) and mature fruits were counted on each of the marked individuals.

### Sampling on an individual basis

In August 2011, we randomly marked 35 focal individuals in each of populations 2 (intermediate population density) and 37 (low population density), with the focal plants separated from each other by more than 1 m. For each focal plant, the number of leaves, flowers (flower scars) and mature fruits were counted. The effect of NND was quantified by measuring the distance from each focal plant to the nearest conspecifics. The effect of LNS was quantified by counting the number of conspecifics within radii of 0.1 m, 0.2 m, and 0.3 m around each focal plant. We included areas with multiple radii because the effects of LNS can be scale-dependent (Roll et al., [1997]).

### Statistical analyses

#### Effects of population size and density on reproduction

To understand the effects of population size and mean population density, we first calculated the population means of leaf number per plant (plant size), flower number per plant, fruit number per plant, and fruit set (fruits/flowers) per plant, in both 2010 and 2011. We first used linear regression analysis to evaluate the effects of population size and mean population density on mean population fruit number or fruit set for each year separately. Because the population means of plant size were highly correlated with mean population flower number (2010: *r*_p_ = 0.865, *P* < 0.001, *n* = 34; 2011: *r*_p_ = 0.861, *P* < 0.001, *n* = 35), fruit number (2010: *r*_p_ = 0.782, *P* < 0.001, *n* = 34; 2011: *r*_p_ = 0.709, *P* < 0.001, *n* = 35) and fruit set in 2010 (*r*_p_ = 0.583, *P* < 0.001, *n* = 34), we used a multiple regression analysis to analyze whether population size and mean population density had an influence on mean population fruit number in 2010 and 2011 or on fruit set in 2010, when accounting for mean population plant size. All variables except mean population fruit set were natural-log-transformed to stabilize variances. For all analyses, we examined the residuals for departures from normality (Shapiro-Wilk test).

### Effects of NND and LNS on reproduction

We first used analyses of covariance to analyze fruit number and fruit set of selected individuals as a function of population (random factor) and NND (covariate). Because individual leaf number (plant size) was highly correlated with individual flower number (population 2: *r*_p_ = 0.750, *P* < 0.001, *n* = 35; population 37: *r*_p_ = 0.829, *P* < 0.001, *n* = 35), individual fruit number (population 2: *r*_p_ = 0.818, *P* < 0.001, *n* = 35; population 37: *r*_p_ = 0.795, *P* < 0.001, *n* = 35) and individual fruit set (population 2: *r*_p_ = 0.531, *P* = 0.001, *n* = 35; population 37: *r*_p_ = 0.570, *P* < 0.001, *n* = 35), we then included individual plant size as a covariate in each of the analyses to evaluate whether NND had effects on fruit number or fruit set when differences in individual plant size were accounted for. All variables except fruit set were natural-log-transformed to stabilize variances. For each analysis, initial models included all possible two-way or three-way interactions involving covariates. Non-significant interactions were dropped from the model using backwards elimination. For all analyses, we examined residuals for departures from normality.

Similarly, we used analyses of covariance to analyze fruit number and fruit set as a function of population (random factor) and 0.1 m LNS (covariate), 0.2 m LNS (covariate) and 0.3 m LNS (covariate). We considered 0.2 m LNS and 0.3 m LNS as the number of additional individuals beyond 0.1 m LNS within 0.2 m, and the number of additional individuals beyond 0.2 m LNS within 0.3-m, respectively. Multicollinearity was assessed by inspection of variance inflation factors, which was always ≤ 2.1, indicating that the level of collinearity was not problematic (Quinn and Keough, [2002]). We then included individual plant size as a covariate in each of the analyses to assess whether LNS had effects on fruit number or fruit set when differences in individual plant size were accounted for. Subsequent manipulations of the statistical model were conducted as described previously for NND. All analyses were conducted using SPSS 17.0.

## Results

### Effects of population size and density on reproduction

Population means for leaf number per plant varied from 6.5 to 13.0 in 2010 and 7.1 to 11.7 in 2011; population means for flower number per plant varied from 6.5 to 22.2 in 2010 and 9.4 to 32.8 in 2011; population means for fruit number per plant varied from 1.7 to 13.1 in 2010 and 3.0 to 11.9 in 2011. Finally, population means for fruit set per plant varied from 0.14 to 0.59 in 2010 and 0.19 to 0.57 in 2011. All measured traits differed significantly among populations within the same year (*P* < 0.001; all variables except fruit set were log-transformed). All correlations were conducted using these log-transformed variables, where applicable.

**Fig. 1:**
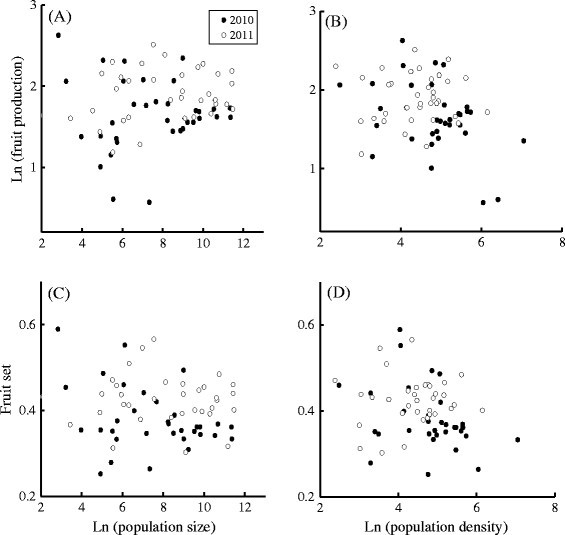
Effects of population size and density on reproduction in *C. agristis*. Relationships between population size and (**a**) mean population fruit production, (**c**) mean population fruit set, and between mean population density and (**b**) mean population fruit production and (**d**) mean population fruit set. Effects of plant size were not accounted for

Mean population fruit number was not correlated to population size in either 2010 (*F*_1,32_ = 0.036, *P* = 0.850) or 2011 (*F*_1,33_ = 1.697, *P* = 0.202) (Fig. 1a). When mean population plant size was included in the regression analysis of each year, mean population fruit number was not related to population size in either 2010 (*t*_31_ = − 0.626, *P* = 0.536) or 2011 (*t*_32_ = 0.066, *P* = 0.948). Meanwhile, mean population fruit number was negatively correlated to mean population density in 2010 (*b* ± *s*_b_ = − 0.192 ± 0.075, *R*^2^ = 0.169, *F*_1,32_ = 6.500, *P* = 0.016), and was not significantly correlated to mean population density in 2011 (*F*_1,33_ = 0.076, *P* = 0.785) (Fig. 1b). When mean population plant size was included in the regression analysis for each year, mean population fruit production was not correlated to mean population density in 2010 (*t*_31_ = − 0.785, *P* = 0.439) or 2011 (*t*_32_ = 0.321, *P* = 0.750). Furthermore, mean population plant size was negatively correlated to mean population density) in 2010 (*b* ± *s*_b_ = − 0.064 ± 0.024, *R*^2^ = 0.180, *F*_1,32_ = 7.037, *P* = 0.012).

Mean population fruit set was not related to population size in either 2010 (*F*_1,32_ = 1.483, *P* = 0.232) or 2011 (*F*_1,33_ = 0.355, *P* = 0.555) (Fig. 1c). When mean population plant size was included in the regression analysis for 2010, mean population fruit set was not correlated to population size (*t*_31_ = − 1.700, *P* = 0.099). Mean population fruit set was negatively correlated to mean population density in 2010 (*b* ± *s*_b_ = − 0.038 ± 0.014, *R*^2^ = 0.186, *F*_1,32_ = 7.305, *P* = 0.011), and not correlated to mean population density in 2011 (*F*_1,33_ = 0.701, *P* = 0.408) (Fig. 1d). When mean population plant size was included in the regression analysis for 2010, the correlation between mean population fruit set and mean population density became non-significant (*t*_31_ = − 1.436, *P* = 0.161).

### Effects of NND

Individual plants of populations 2 and 37 respectively produced an average of 10.3 (SD = 2.6, range = 7 – 18) and 10.6 (SD = 2.5, range = 7 – 17) leaves, and 10.5 (SD = 5.6, range = 2 – 29) and 11.8 (SD = 7.9, range = 3 – 29) fruits. Mean fruit set of individual plants was 0.45 (SD = 0.13, range = 0.25 – 0.70) and 0.47 (SD = 0.12, range = 0.23 – 0.71) in populations 2 and 37 respectively. All of these measured traits did not differ significantly between populations (*P* > 0.5 for all comparisons). Mean NND was 0.020 m (SD = 0.019, range = 0.001 – 0.081 m) and 0.044 m (SD = 0.056, range = 0.001 – 0.230 m) in populations 2 and 37 respectively, and differed significantly between populations (*F*_1,68_ = 4.001, *P* = 0.049).

**Fig. 2:**
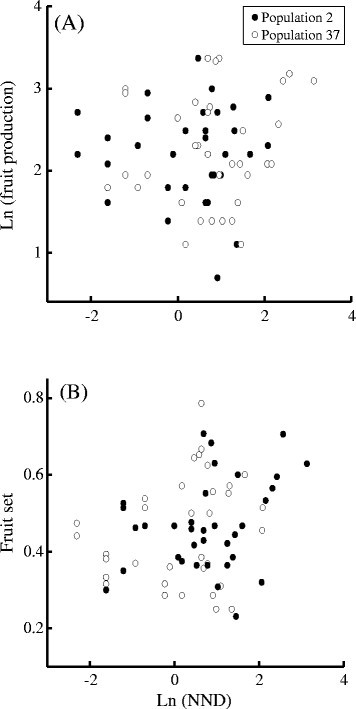
Effects of NND on individual reproduction within populations of *C. agristis.* Relationships between NND and (**a**) individual fruit production and (**b**) individual fruit set. Effects of plant size were not accounted for

Individual fruit number did not vary with NND irrespective of whether or not individual plant size was included in the analysis of covariance (Table 2A-B, Fig. 2a). Individual fruit set was positively correlated with NND (*b* ± *s*_b_ = 0.029 ± 0.013, Table 2A, Fig. 2b). When individual plant size was included in the analysis of covariance, a positive effect of NND on individual fruit set remained (*b* ± *s*_b_ = 0.032 ± 0.011, Table 2B). As expected, individual plant size affected individual fruit number and individual fruit set (Table 2B). Furthermore, individual plant size was not significantly correlated to NND (*F*_1,67_ = 0.150, *P* = 0.699).

**Table 2:** Individual fruit production and fruit set as a function of population, NND and plant size

	Effect	Fruit production	Fruit set
A	Population	*F*_*1,67*_ = 0.006	*F*_*1,67*_ = 0.020
	NND	*F*_*1,67*_ = 0.512	*F*_*1,67*_ = 4.807*
	Model *R*^2^	n.s.	0.069
B	Population	*F*_*1,66*_ = 0.500	*F*_*1,66*_ = 0.063
	NND	*F*_*1,66*_ = 3.015	*F*_*1,66*_ = 9.664**
	Plant size	*F*_*1,66*_ = 125.026***	*F*_*1,66*_ = 33.551***
	Model *R*^2^	0.657	0.383

n.s. = not significant, * *P* < 0.01, ** *P* < 0.01, *** *P* < 0.001

### Effects of LNS

In populations 2 and 37, the mean 0.1 m LNS was 15.8 (SD = 10.6, range = 2–40) and 9.5 (SD = 10.0, range = 1–56), respectively. Similarly, the mean 0.2 m LNS was 24.3 (SD = 18.8, range = 3–72) and 12.3 (SD = 11.3, range = 1–45), respectively, while the mean 0.3 m LNS was 28.3 (SD = 28.0, range = 4–137) and 18.6 (SD = 24.5, range = 1–131), respectively. All pairwise contrasts between populations were significant (*P* < 0.05).

**Fig. 3:**
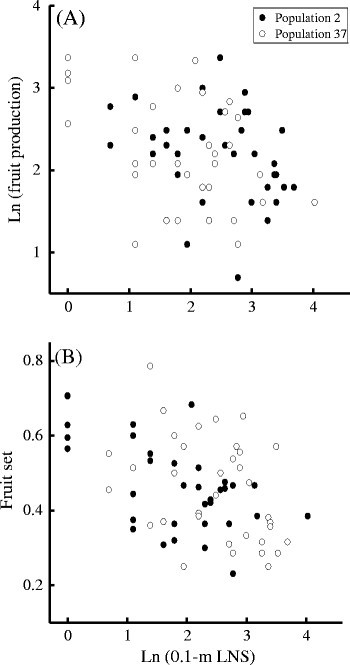
Effects of 0–0.1 m LNS on individual reproduction within populations of *C. agristis.* Relationships between 0.1-m LNS and (**a**) individual fruit production and (**b**) individual fruit set. Effects of plant size were not accounted for

Individual fruit production was negatively correlated to 0.1 m LNS (*b* ± *s*_b_ = − 0.269 ± 0.075, Table 3A, Fig. 3a), and was not correlated to 0.2 m LNS or 0.3 m LNS (Table 3A). When individual plant size was included in the analysis of covariance, individual fruit production was again negatively correlated to 0.1 m LNS (*b* ± *s*_b_ = − 0.127 ± 0.056, Table 3B), and was not correlated to 0.2 m LNS or 0.3 m LNS (Table 3B). Furthermore, individual plant size was negatively correlated to 0.1 m LNS (*b* ± *s*_b_ = − 0.096 ± 0.032, *F*_1,65_ = 9.133, *P* = 0.004), and was not correlated to 0.2 m LNS (*F*_1,65_ = 3.775, *P* = 0.056) or 0.3-m LNS (*F*_1,65_ = 0.033, *P* = 0.856).

**Table 3:** Individual fruit production and fruit set as a function of population LNS and plant size

	Effect	Fruit production	Fruit set
A	Population	*F*_*1,65*_ = 0.555	*F*_*1,65*_ = 1.391
	0.1 m LNS	*F*_*1,65*_ = 15.213***	*F*_*1,65*_ = 16.637***
	0.2 m LNS	*F*_*1,65*_ = 2.807	*F*_*1,65*_ = 0.162
	0.3 m LNS	*F*_*1,65*_ = 1.019	*F*_*1,65*_ = 0.691
	Model *R*^2^	0.196	0.244
B	Population	*F*_*1,64*_ = 2.227	*F*_*1,64*_ = 2.289
	0.1 m LNS	*F*_*1,64*_ = 5.252*	*F*_*1,64*_ = 7.445**
	0.2 m LNS	*F*_*1,64*_ = 0.055	*F*_*1,64*_ = 0.468
	0.3 m LNS	*F*_*1,64*_ = 1.924	*F*_*1,64*_ = 0.731
	Plant size	*F*_*1,64*_ = 102.676***	*F*_*1,64*_ = 23.627***
	Model *R*^2^	0.691	0.448

* *P* < 0.05, ** *P* < 0.01,*** *P* < 0.001

Individual fruit set was negatively correlated to 0.1 m LNS (*b* ± *s*_b_ = − 0.068 ± 0.015, Table 3A, Fig. 3b), and was not correlated to 0.2 m LNS or 0.3 m LNS (Table 3A). When individual plant size was included in the analysis of covariance, individual fruit set was again negatively correlated to 0.1 m LNS (*b* ± *s*_b_ = − 0.042 ± 0.015, Table 3B), and was not correlated to 0.2 m LNS or 0.3 m LNS (Table 3B).

## Discussion

### Effects of habitat fragmentation on reproduction at the population level

The effects of population size on reproduction have been widely studied (Ghazoul, [2005]; Aguilar et al., [2006]; Leimu et al., [2006]). There is a large body of evidence indicating that plants of small populations have lower fruit or seed production than those of large populations because of insufficient pollen quantity and poor pollen quality (e.g. Ågren [1996]; Tomimatsu and Ohara, [2002]; Brys et al., [2004]; Waites and Ågren, [2004]; Hermansen et al., [2014]). However, we did not detect such a pattern in *C. agristis* in either of the years studied, possibly because pollen quantity and quality are not related to its population size. No relationships between fruit or seed number per plant and population size have been reported in some studies (e.g. Alexandersson and Ågren, [1996]; Molano-Flores et al. [1999]; Spigler and Chang, [2008]), suggesting that positive relationships between population size and either pollination or reproduction maybe not the rule. Alternatively, a lack of population size effects might arise if studies examining the effects of population size require the sampling of a wide range of population sizes. This explanation cannot be applied to our results, given that our study included, in both years of study, both populations with only a few individuals and populations with about 100, 000 individuals (Table 1).

In contrast to the vast majority of studies reporting a positive correlation between fruit or seed number per plant and population density (Ghazoul, [2005]; Wagenius, [2006]; Brys et al., [2008]; Feldman, [2008]), the effects of population density on mean population fruit number and fruit set in *C. agristis* varied between years, from negative in 2010 to neutral in 2011. As indicated in Table 1, Fig. 1b and Fig. 1d, some populations (9, 26 and 29) surveyed in 2010 were very dense, and it is possible that a negative correlation between population density and mean population fruit number or fruit set for *C. agristis* may be detectable only when dense populations are surveyed. Our findings support the idea that a 1-year study may not provide a realistic view of the effects of fragmentation on plant reproduction (Hobbs and Yates, [2003]). However, when population plant size was accounted for, no relationship between population density and mean population fruit production or fruit set was evident in either year. These results suggest that the effects of population density on mean population fruit production and fruit set in 2010 were exerted indirectly via a negative effect on population plant size, and that pollination was independent of population density. A negative relationship between population density and mean population plant size might arise if competition for resources increases with density, reducing resource availability for plants in populations of high density (Harper, [1977]).

### Effects of habitat fragmentation on reproduction at the individual level within populations

Many studies have shown that the reproductive success of individual plants may decrease in response to increasing NND or decreasing LNS due to pollen limitation (Allison, [1990]; Roll et al. [1997]; Jakobsson et al., [2009]; Caraballo-Ortiz et al., [2011]). Contrary to these studies, individual fruit set of *C. agristis* in our study increased in response to increasing NND and decreased with increasing LNS at the 0.1 m spatial scale, and these relationships remained even when individual plant size was accounted for. These results are highly suggestive of intra-specific competition for resources: competition for resources decreased with increasing NND and increased with increasing LNS at the 0.1 m spatial scale during fruit maturation. Individual fruit production in *C. agristis* decreased with increasing LNS at the 0.1 m spatial scale but did not change in response to NND when individual plant size was accounted for; this indicates that competition for resources between the focal individual and the nearest conspecific individual may have been too weak to affect fruit production in the focal plants. Similarly, individual plant size of *C. agristis* decreased with increasing LNS at the 0.1 m spatial scale but did not change in response to NND, suggesting that intraspecific competition for resources between neighboring plants during growth was not strong enough to be detected when only the nearest conspecific was considered. These results also suggest that plants of *C. agristis* with fewer neighbors within a 0.1 m radius would benefit from not only a direct negative effect of LNS on reproductive success, but also from an indirect negative effect of LNS on reproductive success, namely via its effect on plant size. This is because large individuals typically have a higher fruit set than small individuals. However, these effects were not evident at the 0.2 m and 0.3 m spatial scales, suggesting that competition for resources in *C. agristis* occurs on very local scales.

## Conclusions

The results of our study indicate that the reproductive success of *C. agristis* does not respond negatively to habitat fragmentation. Although previous studies on both agricultural and experimental populations have shown that high plant density may reduce fruit or seed production through competition and/or plant size (Harper, [1977]; Antonovics and Levin, [1980]; Weiner, [1982]; Mustajärvi et al., [2001]), our study is an instance of negative density-dependent reproduction occurring in a rare and endangered species in the wild. Furthermore, our findings highlight the view that any studies of the effects of fragmentation on reproduction should consider multiple spatial scales (Wagenius, [2006]; Gunton and Kunin, [2007]; Spigler and Chang, [2008]).

Our study has implications for the conservation and management of *C. agristis*. First, our finding that *C. agristis* populations are distributed patchily on forest floors after fragmentation suggests that the maintenance of *C. agristis* habitat should be a priority. Second, small and sparse populations are as important as large and dense populations from a conservation perspective, because the former will be at least as successful, if not more, at reproduction, compared to large and dense populations. Third, a program aimed at re-establishing populations of this species should consider increasing the spacing between plants (compared to populations in the wild), which may reduce competition for resources and thereby increase fruit production.

## Authors’ original submitted files for images

Below are the links to the authors’ original submitted files for images. Authors’ original file for figure 1 Authors’ original file for figure 2 Authors’ original file for figure 3
