# Supplementary figures and images for: Scale-dependent effects of habitat fragmentation on reproduction in the annual Circaeaster agristis, a narrow endemic and threatened species
Source: Bot Stud. 2015 Jun 2;56:15. doi: 10.1186/s40529-015-0095-5 (PMC5434749; doi:10.1186/s40529-015-0095-5)

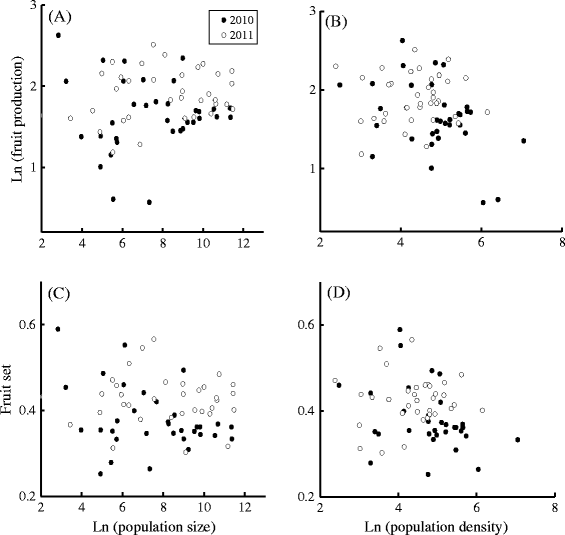

Supplement: Supplementary file 1 — Authors’ original file for figure 1 [file 40529_2015_95_MOESM1_ESM.gif]

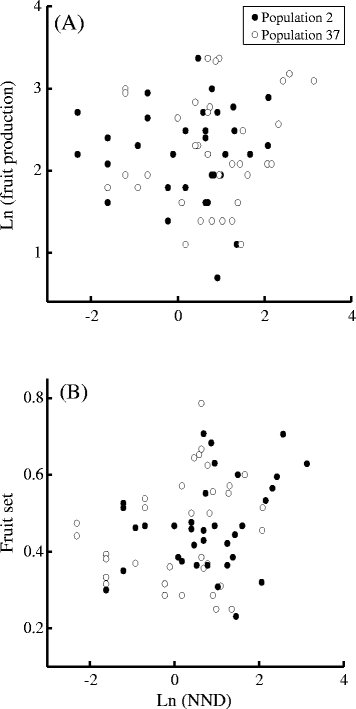

Supplement: Supplementary file 2 — Authors’ original file for figure 2 [file 40529_2015_95_MOESM2_ESM.gif]

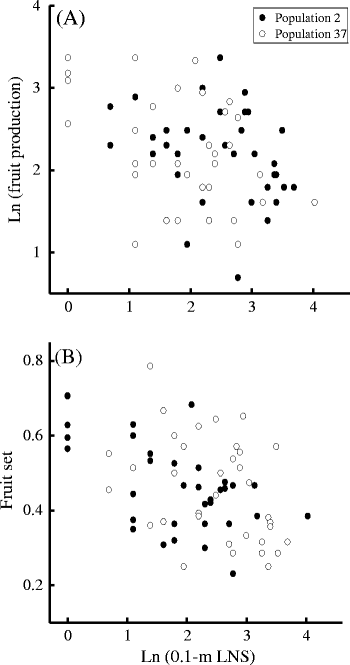

Supplement: Supplementary file 3 — Authors’ original file for figure 3 [file 40529_2015_95_MOESM3_ESM.gif]
